# Testing a cognitive model to predict posttraumatic stress disorder following childbirth

**DOI:** 10.1186/s12884-016-1194-3

**Published:** 2017-01-14

**Authors:** Lydia King, Kirstie McKenzie-McHarg, Antje Horsch

**Affiliations:** 1 The Oxford Institute of Clinical Psychology Training, Isis Education Centre, Warneford Hospital, Oxford, OX3 7JX UK; 2 Department of Clinical Health Psychology, Warwick Hospital, Lakin Rd, Warwick, CV34 5BW UK; 3 Department of Maternal and Child Health, University Hospital Lausanne, Avenue Pierre-Decker 2, CH-1011 Lausanne, Switzerland

**Keywords:** Childbirth, Postnatal, Cognitive model, Traumatic, PTSD, Cognitive predictors, Risk factors

## Abstract

**Background:**

One third of women describes their childbirth as traumatic and between 0.8 and 6.9% goes on to develop posttraumatic stress disorder (PTSD). The cognitive model of PTSD has been shown to be applicable to a range of trauma samples. However, childbirth is qualitatively different to other trauma types and special consideration needs to be taken when applying it to this population. Previous studies have investigated some cognitive variables in isolation but no study has so far looked at all the key processes described in the cognitive model. This study therefore aimed to investigate whether theoretically-derived variables of the cognitive model explain unique variance in postnatal PTSD symptoms when key demographic, obstetric and clinical risk factors are controlled for.

**Method:**

One-hundred and fifty-seven women who were between 1 and 12 months post-partum (*M* = 6.5 months) completed validated questionnaires assessing PTSD and depressive symptoms, childbirth experience, postnatal social support, trauma memory, peritraumatic processing, negative appraisals, dysfunctional cognitive and behavioural strategies and obstetric as well as demographic risk factors in an online survey.

**Results:**

A PTSD screening questionnaire suggested that 5.7% of the sample might fulfil diagnostic criteria for PTSD. Overall, risk factors alone predicted 43% of variance in PTSD symptoms and cognitive behavioural factors alone predicted 72.7%. A final model including both risk factors and cognitive behavioural factors explained 73.7% of the variance in PTSD symptoms, 37.1% of which was unique variance predicted by cognitive factors.

**Conclusions:**

All variables derived from Ehlers and Clark’s cognitive model significantly explained variance in PTSD symptoms following childbirth, even when clinical, demographic and obstetric were controlled for. Our findings suggest that the CBT model is applicable and useful as a way of understanding and informing the treatment of PTSD following childbirth.

## Background

Although childbirth is usually considered a positive life event, it is now recognised that labour and delivery have the potential to fulfil the traumatic stressor criteria as defined in the Diagnostic and Statistical Manual (fifth edition; DSM 5 [1]). It is suggested that between 20 and 30% of women experience childbirth as traumatic [2–4]. Approximately 10% of women may have a severe traumatic stress response in the initial postpartum weeks, but only between 0.8 and 6.9% of women will go on to develop posttraumatic stress disorder (PTSD) as a direct result of childbirth events [2].

Not only is postnatal PTSD highly distressing for the mother, it can also impact negatively on her attachment with her child [5, 6] and her relationship with her partner [7, 8]. Furthermore, the presence of PTSD during a subsequent pregnancy can have an impact on birth weight and gestational age of the infant [9, 10].

A range of perinatal risk factors that may increase women’s vulnerability to developing PTSD symptoms following childbirth has been established [2, 11–14]. *Pretraumatic* risk factors include fear of childbirth which could include both a primary tokophobia presentation (fear of childbirth in women who have not previously delivered) or a secondary tokophobia presentation (fear of childbirth in women who have experienced a previous, traumatic delivery; e.g., [15]), psychiatric history (especially previous PTSD), psychiatric difficulties during pregnancy and previous trauma (including previous traumatic childbirth or sexual abuse; [2, 11]. Factors such as parity, socioeconomic status, age, ethnicity and unplanned pregnancy have shown associations with PTSD symptoms, although their influence can be small and inconsistent [11]. *Intrapartum* risk factors thought to be especially important are subjective distress in labour (including pain, fear, dissociation during birth, but especially loss of control and negative emotions about childbirth) and obstetric factors such as emergency caesarean sections and instrumental deliveries [11, 16]. Waldenstrom et al. [17] highlight the particular impact of unexpected complications during the labour/delivery experience as having a significant impact. Severe infant complications and low support during childbirth are also associated with PTSD [11]. For example, Pierrehumbert et al. [18] examined the specific infant complication of premature delivery, drawing a clear link with impact on parental PTSD.

*Postnatal* factors that predict the development of PTSD have been less well studied. However, coping style, additional stress and in particular low social support appear to be associated with PTSD [2]. For example, a lack of partner support has been identified as a risk factor for the development of PTSD following childbirth [7] and good social support has been found to be protective against the development of PTSD following trauma in other populations (e.g., [19]). Postnatal factors may be of particular clinical importance, as they may be the most amenable to change in order to prevent the development or maintenance of PTSD.

Although research into PTSD following childbirth is increasing and risk factors are becoming clearer, the literature lacks a strong theoretical basis [20] and there is limited understanding of the postnatal factors that may help to maintain traumatic stress responses. The cognitive model proposed by Ehlers and Clark [21] is widely used in the formulation and treatment of PTSD in other adult populations. It suggests that a sense of current threat is produced by negative cognitive appraisals both during and after the traumatic event. This threat, in conjunction with a fragmented and poorly integrated traumatic memory, can be unintentionally triggered by situations resembling some aspect of the traumatic event. PTSD is then maintained through unhelpful cognitive (e.g. thought suppression) and behavioural (e.g. reminder avoidance) strategies. Despite these strategies being aimed at controlling the sense of threat/symptoms, they can directly produce symptoms and/or prevent change in negative appraisals or the nature of the trauma memory. All components of the model have received empirical support in the general PTSD literature (for a review, see [22]).

Evidence supports the utility of the cognitive model in understanding and treating PTSD in adults, but the qualitative difference between childbirth and what we traditionally consider to be a traumatic experience needs to be considered when evaluating its applicability to this population. Culturally, childbirth and motherhood are associated with positive expectations [23]. Women can perceive the birth of their baby as a positive outcome even if the birth is appraised as traumatic [24]. Women may have hopes and expectations for the process of childbirth that contrast with their actual experience [25]. There are at least two individuals involved in any childbirth experience (mother and child) and the woman might fear for her own life and/or that of her (unborn) baby [26]. Following a traumatic birth the mother is expected to form a close relationship with her baby, even though the baby may be a reminder of the traumatic experience [26]. Ayers et al. [27] found that for mothers developing PTSD following a traumatic childbirth experience, the mother-baby bond was significantly affected, and that in the longer term many of these infants appeared to be insecurely attached, highlighting the importance of this issue. Additionally, memory processes may be impacted by the use of systemic analgesic medication [28]. It is therefore important to investigate if the cognitive model also applies to the childbirth population.

Previous studies have investigated single cognitive variables in isolation, such as memory disorganisation [28], negative cognitive appraisals of the birth and its sequelae [29] and perinatal dissociation [3]. Two studies have more systematically examined cognitive predictors in a childbirth population. Ford and colleagues [29] found that prior experiences, beliefs, and coping state as well as negative appraisals of childbirth explained 23% of variance in posttraumatic stress symptoms at 3 weeks post-birth. Vossbeck-Elsebusch et al. [30] reported that peritraumatic dissociation, negative appraisals, thought suppression, rumination, and perseverative thinking accounted for an additional 23% of variance of PTSD symptoms after accounting for known risk factors. However, no study has so far looked at all the key processes described in the cognitive model. More comprehensive investigation of the cognitive model, including elements such as the nature of the trauma memory, peritraumatic processing (peritraumatic dissociation, perceptual processing, lack of self-referent processing), negative appraisals of trauma and/or its sequalae, and the use of dysfunctional cognitive and behavioural strategies (thought suppression, rumination, and numbing) may further our understanding of the applicability of the cognitive model in the development and maintenance of PTSD following traumatic childbirth.

This study therefore aimed to investigate whether theoretically-derived variables of the cognitive model explain unique variance in postnatal PTSD symptoms when key demographic, obstetric and clinical risk factors are controlled for. It was hypothesised that (1) Demographic, obstetric and clinical risk factors will explain some variance in PTSD symptoms; (2) Cognitive and behavioural factors derived from the Ehlers and Clark model [21] will explain some variance in PTSD symptoms; and (3) Cognitive and behavioural factors will explain additional unique variance in PTSD symptoms when clinical, obstetric and demographic risk factors are controlled for.

## Method

### Sample

Women were eligible to participate in this cross-sectional study if they had given birth to a live and healthy baby and were between one and 12 months post childbirth. Women were excluded if their baby had been cared for in a special care baby unit or a neonatal intensive care unit for more than four hours post childbirth, if their level of English was not sufficient to be able to complete the questionnaires or if they were under the age of 18. Data collection took place between December 2013 and May 2014.

In total, 325 women accessed the online survey. Of the 167 who did not complete the survey, 44 individuals appeared not to have met the inclusion criteria and 123 did not fully complete the survey for unknown reasons. One case was identified as ineligible as it was completed in reference to a previous birth. This left a total sample of 157 (48.31%) individuals who completed the survey sufficiently for inclusion in the analysis. The descriptive characteristics of the sample for cognitive, demographic and obstetric variables are summarised in Table 1. Ninety-four percent of women were of white ethnic origin and the age range was 18 to 44 years (*M* = 29.82, *SD* = 5.13). The modal income range was £30,000–£39,999 but women were well represented from a wide range of incomes. On average, women were 194 days post birth (approximately 6.5 months) when they completed the survey. One third of women (33.8%) reported having experienced mental health difficulties prior to pregnancy whereas 19% of women reported having experienced mental health difficulties during their most recent pregnancy.

**Table 1:** Demographic, clinical and obstetric data of the sample

Demographic data (*n* = 157ª)	Mean(SD) *f*(%)
Maternal age (*n* = 119)	29.82 (5.13)
Ethnicity	
White UK	134 (85.4%)
White Irish	3 (1.9%)
White other	10 (6.4%)
Black African	2 (1.3%)
Chinese	2 (1.3%)
Pakistani	2 (1.3%)
Other	4 (2.5%)
Occupation	
Full-time	75 (47.8%)
Part-time	34 (21.7%)
Student	5 (3.2%)
Home-maker	43 (27.4%)
Family income	
£0–£9,999	13 (8.3%)
£10,000–£19,999	28 (17.8%)
£20,000–£29,999	22 (14.0%)
£30,000–£39,999	32 (20.4%)
£40,000–£49,999	15 (9.6%)
£50,000–£59,999	17 (10.8%)
£60,000–£69,999	10 (6.4%)
£70,000 +	20 (12.7%)
Marital status	
Single	4 (2.5%)
In a relationship	13 (8.3%)
Living with partner	46 (29.3%)
Married	94 (59.9%)
Clinical risk factors (*n* = 157)	Mean(SD), or *f*(%)
History of traumatic experiences	
Yes	54 (34.6%)
No	103 (65.6%)
Psychiatric history	
Yes	53 (33.8%)
No	104 (66.2%)
Mental health problems during preg	
Yes	30 (19.1%)
No	127 (80.9%)
Mental health problems since birth	
Yes	41 (26.1%)
No	116 (73.9)
Social support (MOSSSS)	80.18 (15.42)
Emotional/informational	33.6 (6.43)
Tangible	17.24 (3.24)
Affectionate	12.37 (3.09)
Social interaction	12.85 (2.73)
Obstetric variables (*n* = 157)	Mean(SD), or *f*(%)
Mode of delivery	
Vaginal	106 (67.5%)
Instrumental	29 (18.5%)
Planned C-section	6 (3.8%)
Emergency C-section	16 (10.2%)
Episiotomy	
Yes	26 (16.6%)
No	131 (83.4%)
Pregnancy complication	
Yes	45 (28.7%)
No	
Mother complications	
Yes	47 (29.9%)
No	70 (70.1%)
Infant complications	
Yes	26 (16.6%)
No	131 (83.4%)
CEQ	
Own capacity	20.71 (5.13)
Professional support	16.85 (3.97)
Perceived safety	17.59 (4.1)
Participation	9.23 (2.57)
Total	64.37 (12.54)
Clinical self-report measures (*n* = 157)	Mean(SD)
EPDS	7.01 (5.5)
TES	
Arousal	9.14 (3.85)
Avoidance/numbing	7.10 (2.90)
Re-experiencing	5.18 (2.33)
Total	21.42 (7.89)

*SD* standard deviation, *f* frequency, *EPDS* Edinburgh postnatal depression scale, *CEQ* childbirth experience questionnaire, *TES* trauma experience survey

ªunless otherwise stated

### Procedure

Questionnaires were hosted on a survey website using Qualtrics, which allowed women to participate anonymously online. Women were recruited through a variety of online and paper sources. Online advertisements were placed on social media sites, posted in social media groups and in relevant online forums. Poster and leaflet advertisements were displayed in areas frequented by mothers in the local community (e.g. community centres, mother and baby groups). Advertisements provided a link which directed women to the Oxford Perinatal Psychology Research Group where full details of the study were available. From this website, participants were able to download a printable copy of the participant information sheet, organisation contact details, and directly access the online survey, which contained the consent form and list of eligibility criteria. Women gave their informed consent before participating; they were automatically directed to an exit page if any of the consent items were not endorsed. A list of organisations offering support to mothers and families following a range of difficulties was provided both at the beginning and end of the survey with relevant contact information.

### Measures

#### Psychopathological symptoms

##### Posttraumatic stress disorder symptoms

As the DSM-5 had only been recently published before data collection took place, no validated PTSD measures were available that corresponded with the new criteria. We therefore used the Trauma Experience Survey (TES), a 23-item questionnaire designed in accordance with the DSM-IV-TR [31] and adapted for use with a postnatal population [26]. Four items assess the criterion A and are rated from 0 = *not at all* to 4 = *very much*. Criterion A is fulfilled if items 1, 2 and/or 3, and 4 are marked as 3 or 4. Following this, 17 items assess the frequency of PTSD symptoms and are rated from 0 = *never/not at all* to 4 = *often*. After that, the severity of each symptom is assessed on a scale from 0 = *not at all* to 10 = *extremely influenced in daily life*. Finally, the duration of symptoms is assessed by a 13-point scale ranging from “*less than 4 weeks*” to “*more than 12 months*”. The questionnaire gives an overall score of PTSD symptom frequency and can estimate if a person is likely to fulfil criteria for a diagnosis of PTSD. It has good psychometric properties [26]. Due to the low levels of PTSD caseness predicted by the literature, the measure was chosen to primarily measure symptom frequency. For the current study, Cronbach’s alpha reliability coefficients for the subscales were .90 (re-experiencing) .79 (avoidance and numbing) .87 (hyperarousal) and .81 for the total.

##### Depressive symptoms

The Edinburgh Postnatal Depression Scale (EPDS) is a 10-item screening questionnaire [32] with good psychometric properties [33]. It specifically excludes normal postnatal symptoms which could be interpreted as depressive symptomatology. Each item is scored from 0 to 3. Total scores can range from 0 to 30 with higher scores indicating more depressive symptoms. A score of 10 indicates possible minor depression and a score of 13, possible major depression. The Cronbach alpha for this study was .82.

#### Demographic, obstetric and clinical risk factors

##### Childbirth experience

The Childbirth Experience Questionnaire (CEQ [34]) is a 22-item questionnaire with good psychometric properties that measures women’s perceptions of their labour and childbirth. Factor analysis identifies four domains: own capacity, which includes questions regarding sense of control, personal feelings during childbirth and pain; professional support, including items about information and midwifery care; perceived safety, containing questions relating to sense of security and memories from the childbirth; and participation containing items regarding the opportunities a woman had to influence the birthing process. For the current study, Cronbach’s alpha reliability coefficients for the subscales were .87 (own capacity), .93 (professional support), .81 (perceived safety), .80 (participation) and .79 for the total.

##### Postnatal social support

The Medical Outcomes Study Social Support Survey (MOSSSS) is designed to comprehensively measure various dimensions of social support [35]. Participants answer how often 20 types of support are available to them from five options ranging from “none of the time” to “all of the time”. It has four functional support scales including: emotional/informational, tangible, affectionate and positive social interaction. All subscales have good reliability, are fairly stable over time and show fair construct validity [35]. For the current study, Cronbach’s alpha reliability coefficients for the subscales were .92 (emotional/informational support), .88 (tangible support), .89 (affectionate support), .88 (positive social interaction) and .89 for the total.

##### Demographic, mental health and obstetric information

This included demographic questions about age, ethnicity, income, and marital status. It also included questions on obstetric history, parity, information about the birth and any complications. Questions about previous trauma experiences and history of mental health difficulties prior to or during the most recent pregnancy were included as well as Likert scales asking women to rate how satisfied they were with birth partner support (“If you had a friend or family member support you during childbirth, as a whole, how well supported did you feel by them during labour and childbirth? Please leave blank if this question is not applicable to you.” rated from 0 = *completely unsupported* to 100 = *completely supported*) and whether they experienced fear prior to childbirth (“Before or during your most recent pregnancy, did you experience intense and distressing fear about the upcoming labour and childbirth?” rated from 0 = *no fear/only slight apprehension* to 100 = *intensely distressing fear*).

#### Cognitive and behavioural predictors

##### Nature of trauma memory

The 16-item Trauma Memory Questionnaire (TMQ) contains three subscales that measure the nature of the trauma memory: deficits in intentional recall (the extent to which the trauma memory is incomplete or disorganised, e.g., “I feel that my memory for labour and childbirth is incomplete.”), intrusions (the extent to which memories have strong perceptual elements, are easily triggered or accompanied by sense of reliving, e.g., “When I remember the labour/childbirth it is like it is happening again, here and now.”), and negative appraisals of deficits in recall (e.g, I find my inability to remember things about childbirth frustrating/distressing.”). Participants gave their response to each item on a five-point Likert scale (0 = *not at all* to 5 = *very strongly*). It has good reliability [36, 37]. In the current study, Cronbach’s alpha reliability coefficients for subscales were .89 (memory disorganisation), .80 (negative appraisal of deficit in intentional recall), .89 (intrusions) and .40 for the total. This indicates that the total score should not be used in this sample.

##### Peritraumatic processing

Three questionnaires with good psychometric properties were used to assess peritraumatic processing [36, 37]. The 9-item State Dissociation Questionnaire (SDQ [38]) which measures peritraumatic dissociative experiences such as derealisation, depersonalisation, detachment, altered time sense and emotional numbing. The 8-item Data-Driven Processing Scale (DDPS; (Ehlers: Data-driven versus conceptual processing questionnaire, unpublished manuscript)) assesses the extent to which a person is engaged with surface level perceptual processing during a traumatic experience. Finally, the 8-item Lack of Self-Referent Processing Scale (LSRPS [37]) assesses the extent to which a person processed a traumatic experience as happening to themselves and incorporated the experience with other autobiographical information. For the current study, Cronbach’s alpha reliability coefficients were .88 (SDQ), .88 (DDPS), and .81 (LSRPS).

##### Negative appraisals of trauma and/or its sequelae

The Posttraumatic Cognitions Inventory (PTCI [39]) is a 36-item measure of trauma-related thoughts and beliefs. Questions are answered on a 7-point Likert scale (“totally agree” to “totally disagree”). The scale assesses three dimensions of traumatic thoughts: negative cognitions about the self (NCATS; including questions regarding general negative self-view, permanent change, alienation, hopelessness, self-trust and negative interpretation of symptoms), negative cognitions about the world (NCATW; including questions regarding an unsafe world and mistrust of other people) and self-blame. Adequate psychometric properties in the postnatal population were shown [29]. For the current study, Cronbach’s alpha reliability coefficients were calculated to be .94 (NCATS), .89 (NCATW), .71 (self-blame) and .65 for the total.

##### Dysfunctional cognitive and behavioural strategies

The Response to Intrusions Questionnaire (RIQ; [40, 41]) is a 19-item inventory that assesses the use of dysfunctional cognitive and behavioural strategies in response to intrusions of the trauma, which was validated in a perinatal population [30]. It contains three subscales: efforts to suppress thoughts and intrusions, rumination and numbing (detaching or dissociation when memories occur). For the current study, Cronbach’s alpha reliability coefficients for the subscales were .93 (thought suppression), .91 (rumination), .86 (numbing) and .82 for the total.

### Data analysis

The data were analysed using IBM SPSS Statistics 20. Descriptive statistics were first used to describe the sample. All continuous variables were assessed for violations of normality using skewness and kurtosis statistics. For dichotomous variables the distribution of the dependent variable (PTSD symptom frequency measured by TES total score) was assessed across both values of each independent variable. Where assumptions of normality were violated for continuous variables, transformations were performed and normality reassessed. Following this procedure, only seven of the 36 variables fulfilled the assumption of normality. As the TES total score also violated the assumption of normality, non-parametric tests (Mann Whitney U tests, Kruskal-Wallis tests and Spearmans Rho correlations) were used to assess the relationships between all independent variables and the dependent variable. Only those variables that had a significant relationship with the TES total score were included in subsequent regression analyses. Correlation coefficients were calculated between all independent continuous variables and assumptions for regression were assessed. Assumptions of normally distributed residuals were met for each regression analyses and therefore it was acceptable for variables that did not meet assumption of normality to be included. All regression analyses also met assumptions regarding the linearity of relationships, absence of collinearity, and independence of errors. However, visual inspection of a matrix scatterplot identified evidence of heteroscedasticity. This meant that the error terms did not have constant variance as values of the independent variables increased. Log transformations were attempted but heteroscedasticity remained. Regressions performed using data that violates the assumption of homoscedasticity still provides an unbiased estimate of the relationship between the predictor variable and the outcome for the sample, but standard errors and therefore significance values obtained may be biased [42]. Therefore it was decided to proceed with regression analysis but interpret any significance values approaching *p* = .05 with extreme caution. Simultaneous regression was used to answer the first and second hypotheses. Hierarchical stepwise regression was used to test the third hypothesis. Power calculation assuming a medium effect size of 0.15, ∝ error probability = .05 and power (∝- β err prob) = .80 was used was estimated prior to recruitment to give recruitment targets (*n* = 114).

## Results

### Prevalence of PTSD and depression

The mean total symptom TES score was 21.42 (SD = 7.89); 26.1% of women (*n* = 41) met criterion A of DSM-IV, 5.7% of mothers (*n* = 9) were likely to meet all the criteria for a diagnosis of PTSD and 27.4% were partially symptomatic (i.e. met criteria for at least one symptom cluster but not all remaining criteria). The average EPDS score was 7.01 (SD = 5.5) which falls below the level that would indicate probable depression, although 26.8% of women (*n* = 42) scored within a range that did indicate probable depression. Self-report measures indicated that 55.6% (*n* = 5) of those who appeared to meet criteria of PTSD also had EPDS scores in the probable range for depression.

### Demographic, clinical and obstetric risk factors for PTSD symptom frequency

Due to violation of the assumption of normality in TES scores Spearman’s correlation coefficients were conducted to identify whether obstetric, demographic and clinical related variables were related to PTSD symptom frequency. Mann-Whitney U tests were conducted for dichotomous variables and Kruskal-Wallis tests for those with more than one category (Table 2). Obstetric factors significantly related to TES scores included mode of delivery (*p* = .01) and maternal complications (*p* = .001) but not infant complications (*p* = .72), pregnancy complications (*p* = .07) or episiotomy (*p* = .10). Prenatal clinical factors that showed a significant relationship with TES scores were pre-pregnancy psychiatric history (*p* = .01), psychiatric history during pregnancy (*p* = .01), and fear of childbirth (*p* = .01). However trauma history was not related (*p* = .09). Birth related clinical factors significantly related to the TES were support from partner during birth (*p* < .001) and all four subscales of the CEQ including perceived capacity during birth (*p* < .001), professional support (*p* < .001), perceived safety (*p* < .001), and participation in birthing decisions (*p* = .01). Significant relationships were also found with the four subscales measuring types of postnatal social support including: informational/emotional support (*p* < .001), tangible support (*p* < .001), affectionate support (*p* < .001) and social interaction (*p* < .001). The only demographic variable that had a significant relationship with TES scores was marital status (*p* = .05). No relationship was found between TES scores and ethnicity (*p* = .16), occupation (*p* = .40) and family income (*p* = .67).

**Table 2:** Relationships between demographic, obstetric and clinical variables and PTSD symptom frequency

Variable	*r*_*s*_	*U(Z)*	*X* ^*2*^ *(df)*	*p*
Clinical				
Social support (MOSSSS)				
Emotional/informational	− .37	-	-	<.001
Tangible	− .39	-	-	<.001
Affectionate	− .37	-	-	<.001
Social interaction	− .43	-	-	<.001
Fear of childbirth	.22	-	-	.01
Partner support during birth	− .33	-	-	<.001
Childbirth Experience (CEQ)				
Own capacity	− .35	-	-	<.001
Professional support	− .3	-	-	<.001
Perceived safety	− .48	-	-	<.001
Participation	− .22	-	-	.01
Trauma history	-	2322.5(−1.72)	-	.09
Psychiatric history (pre-pregnancy)	-	2093(−2.48)	-	.01
Psychiatric history during pregnancy	-	1321(−2.63)	-	.01
Depressive symptoms (EPDS)	− .02	-	-	.82
Obstetric				
Pregnancy complications	-	2060(−1.80)	-	.07
Maternal complications	-	1708(−3.39)	-	.001
Infant complications	-	1627.5(− .36)	-	.72
Mode of delivery	-	-	11.36(3)	.01
Episiotomy		1362(−1.63)	-	.10
Demographic				
Marital status	-	853(−1.93)		.05
Ethnicity		-	9.21(6)	.16
Occupation	-	-	2.95(3)	.40
Income	-	-	4.89(7)	.67

*r*_*s*_ spearmans rho correlation co-efficient, *U* Mann Whitney *U* test statistic, *Z* standardised test statistic, *X* ^2^ Kruskal-Wallis test statistic, *df* degrees of freedom

A simultaneous multiple regression analysis was conducted to identify whether demographic, obstetric and clinical variables predicted a significant amount of the variance in PTSD symptom frequency. Only variables that had a significant relationship with the TES score (see Table 2) were entered (*n* = 15). These variables were significant predictors of PTSD symptoms, *F*(17,139) = 6.17, *p* < .001, adj. *R* ^2^ = .43, accounting for 43% of the variance. Only three variables were significant individual predictors of PTSD symptoms: use of instruments to assist vaginal delivery (*p* = .03) and low satisfaction with partner support during delivery (*p* = .04), and low level of perceived safety during birth (CEQ) as the most significant predictor (*p* = .004). Regression coefficients and standard errors can be found in Table 3.

**Table 3:** Demographic, obstetric and clinical risk factors of frequency of PTSD symptoms

Variable	PTSD symptom frequency (TES)			
	*β*	SE	*t*	*p*
(Constant)		4.68	10.41	<.001
Marital status (single)	− .13	1.72		
Psychiatric history	.06	1.23	0.86	.39
Psychiatric history (pregnancy)	.07	1.49	0.91	.37
Social support (MOSSSS)				
Emotional/informational	.18	0.18	−0.01	.37
Tangible	.34	0.34	−1.18	.24
Affectionate	.36	0.36	0.89	.38
Positive social interaction	.44	0.44	−1.70	.09
Mode of delivery				
Instrumental delivery	.16	1.49	2.17	.03
C Section in labour	− .02	1.95	−0.29	.78
Planned C section	− .06	2.97	0.83	.41
Fear of childbirth	.04	0.20	0.56	.57
Partner support during birth	− .15	0.26	−2.07	.04
Maternal complications	− .08	1.18	1.17	.24
Childbirth Experience Questionnaire				
Own capacity	.04	0.16	−0.59	.56
Professional support	.02	0.17	0.25	.80
Perceived safety	− .36	0.24	−2.94	.004
Participation during birth	.06	0.27	0.64	.52

*β* regression coefficient, *SE* standard error, *t t*-test statistic, *p* probability

### Cognitive behavioural predictors of PTSD symptom frequency

Spearman’s correlation coefficients were conducted to identify whether cognitive behavioural variables were associated with PTSD symptom frequency. All cognitive variables were significantly positively associated with PTSD symptoms (all *p* < .01; see Table 4).

**Table 4:** Relationships between cognitive behavioural variables and PTSD symptom frequency

Variable	*r*_*s*_
Trauma Memory Questionnaire	
Intentional recall scale	.40***
Intrusions	.38***
Negative Appraisals	.35***
Processing questionnaires	
Data-driven processing	.36***
State Dissociation	.41***
Lack of self-referent processing	.42***
Posttraumatic Cognitions Inventory	
Negative cognitions about the self	.58***
Negative cognitions about the world	.42***
Self-blame	.25**
Response to Intrusions Questionnaire	
Suppression	.57***
Rumination	.56***
Numbing	.49***

*r*_*s*_ Spearmans Rho correlation co-efficient

**= *p*  < .01. *** = *p* < .001

A simultaneous multiple regression analysis was conducted to identify whether cognitive variables predicted a significant amount of the variance in TES score. All cognitive behavioural variables were entered and predicted 72.7% of the variance, *F*(12,144) = 35.58, *p* < .001, adj. R^2^ = .727. Six variables were significant predictors of PTSD symptoms: increased negative cognitions about the self (PTCI; *p* < .001), deficits in intentional recall (RIQ; *p* = .001) and high scores on all three subscales of the RIQ: the suppression subscale (*p* = .03), the rumination subscale (*p* < .001) and the numbing subscale (*p* = .013. In addition, the negative appraisals scale of the TMQ was a significant negative predictor (*p* < .001) of PTSD symptoms despite the two variables having a significant positive correlation. Regression coefficients and standard errors can be found in Table 5.

**Table 5:** Cognitive behavioural predictors of PTSD symptom frequency

Variable	PTSD symptom frequency (TES)			
	*β*	SE	*t*	*p*
(Constant)		1.77	6.03	.001
Trauma Memory Questionnaire				
Intentional recall scale	.20	.10	3.32	.001
Intrusions	− .06	.06	1.01	.28
Negative Appraisals	− .30	.32	−4.9	.001
Processing questionnaires				
Data-driven processing	.03	.40	−0.57	.57
State Dissociation	.09	.38	1.1	.27
Lack of self-referent processing	− .09	.39	−1.45	.15
Posttraumatic Cognitions Inventory				
Negative cognitions about the self	.32	.66	4.39	.001
Negative cognitions about the world	− .01	.35	−0.14	.89
Self-blame	.03	.97	0.69	.49
Response to Intrusions Questionnaire				
Suppression	.16	.17	2.23	.03
Rumination	.40	.11	5.80	.001
Numbing	.19	.26	2.51	.01

*β* regression coefficient, *SE* standard error, *t t*-test statistic, *p* probability

### Risk factors and cognitive behavioural predictors of PTSD symptom frequency

A hierarchical stepwise regression analysis was conducted to investigate whether cognitive and behavioural variables explained variance in PTSD symptom frequency scores over and above the variance predicted by clinical, obstetric and demographic risk factors. The final model (Table 6) included four clinical, obstetric and demographic variables explaining 36.6% of the variance, *F*(4,152) = 23.56, *p* < .001, adj. *R* ^2^ = .37. Only two of the variables were significant predictors: low levels of perceived safety (CEQ; *p* = .05) and lack of postnatal positive social interaction (MOSSS; *p* = .01). Two were included in the final model but were not significant following the second step: the use of instruments during vaginal delivery (*p* = .91), and being single (*p* = .38).

**Table 6:** Variables included in the final stepwise hierarchical regression model

PTSD symptom frequency (TES score)				
Variables included in final model	*β*	SE	*t*	*p*
(Constant)		2.89	7.19	.001
Perceived safety (CEQ)	− .10	0.10	−1.94	.05
Positive social interaction	− .13	0.14	−2.7	.01
Instrumental delivery	.01	0.91	0.11	.91
Single	− .04	1.09	−0.88	.38
Negative cognitions of self (PTCI)	.29	0.54	4.86	.001
Rumination (RIQ)	.36	0.11	5.32	.001
Numbing (RIQ)	.27	0.24	3.75	.001
Deficits in intentional recall (TMQ)	.19	0.09	3.73	.001
Negative appraisals of memory deficits (TMQ)	− .24	0.31	−4.05	.001

*β* regression coefficient, *SE* standard error, *t t*-test statistic, *p* probability

In the next step, five cognitive behavioural factors were added to the model. All had been identified as significant predictors in the previous regression model: high levels of negative cognitions about the self (PTCI; *p* < .001), high levels of rumination (RIQ; *p* < .001), and numbing (RIQ; *p* < .001) in response to intrusions, and deficits in intention recall (TMQ; *p* < .001). Again, it was noted that despite a positive correlation with frequency of PTSD symptoms, negative appraisals of deficits in recall was a negative predictor in this regression (TMQ; *p* < .001). The whole model explained 73.7% of the variance *F*(9,147) = 49.5, *p* < .001, adj. *R* ^2^ = .737 and the additional cognitive behavioural factors explained an additional 37.1% of variance of PTSD symptoms.

## Discussion

This study investigated whether theoretically-derived variables of the cognitive model of PTSD explain unique variance in PTSD symptoms in women following childbirth when key demographic, obstetric and clinical risk factors were controlled for. It was hypothesised that (1) Demographic, obstetric and clinical risk factors will explain some variance in PTSD symptoms; (2) Cognitive and behavioural factors will explain some variance in PTSD symptoms; and (3) Cognitive and behavioural factors will explain additional unique variance in PTSD symptoms when clinical, obstetric and demographic risk factors are controlled for.

Results indicated that when demographic, obstetric and clinical risk factors were considered alone, 43% of variance in PTSD symptoms was explained. Cognitive behavioural predictors alone explained 72.7% of the variance. When cognitive behavioural factors were added into a hierarchical regression with risk factors controlled for, the overall model explained 73.7% of variance, with cognitive behavioural factors explaining 37.1% unique variance in PTSD symptoms. The effect sizes are higher than those estimated in two previous studies [29, 30] investigating only some aspects of the cognitive model in a childbirth population, thus confirming the usefulness of a comprehensive investigation of all key cognitive behavioural components of the cognitive model of PTSD.

We found that 5.7% of the sample met full DSM-IV criteria for PTSD. Although investigation of prevalence was not a key aim for this study as endorsement of PTSD symptoms has been found to be elevated in online samples [43], this estimate is at the higher end of the range suggested by Ayers [2]. Twenty-six percent of the sample fulfilled criterion A suggesting they experienced their childbirth as traumatic, which is consistent with previous findings [29].

Regarding demographic, obstetric and clinical risk factors of PTSD, low levels of perceived safety during birth, low levels of partner support during birth, and the use of instruments to assist vaginal delivery emerged as significant individual risk factors. This replicates previous findings [2, 11, 12]. Two of these remained in the final model of the hierarchical regression: low levels of perceived safety and instrumental delivery. Two new demographic, obstetric and clinical predictors emerged in the final regression: being single and low levels of perceived postnatal social support. However, neither instrumental delivery nor being single was significant in the final model once cognitive behavioural variables were included.

The finding that perceived safety during birth was the strongest significant predictor of PTSD symptom frequency (and remained in the final regression model) is perhaps unsurprising. It is consistent with the literature that identifies mothers’ subjective distress during childbirth and labour as one of the strongest predictors of PTSD [11]. A low level of perceived safety would also be consistent with criterion A of the PTSD diagnostic criteria [31] in that those who experienced fear, anguish, horror or helplessness during childbirth might reasonably be expected to also experience low levels of perceived safety.

The finding that partner support during the birth was a significant predictor is consistent with the existing literature [26, 44], although many studies so far have primarily focused on professional support during birth [45].

Previous reviews have identified obstetric emergency and mode of delivery as strong predictors of PTSD symptoms [2, 11], although this finding has not always been consistent [29, 46]. In the current sample, instrumental delivery was a significant predictor, which has previously been found [47]. The current sample did not include mothers whose babies were cared for in special care baby units or neonatal intensive care unit for longer than four hours. It may be that the failure to find other significant obstetric factors (or indeed infant complications) is due to the inclusion criteria.

The final regression model identified positive postnatal social support as a significant predictor. Other studies have highlighted the importance of social support in predicting PTSD following childbirth [29, 48, 49]. However, our model suggests that it is *positive* social interaction following childbirth that is particularly protective. Alternatively, it is possible that women with higher levels of PTSD symptoms perceive less positive social interaction rather than objectively experience it.

Focusing on cognitive behavioural variables in our study, negative cognitions about the self in relation to the birth were the strongest of the cognitive behavioural predictors in both the original regression and hierarchical regression. These findings confirm that the addition of negative cognitions as a criterion for PTSD in the DSM-5 [1] is appropriate and applicable to the postnatal population.

Regarding dysfunctional cognitive and behavioural strategies, thought suppression, rumination and numbing were significant predictors in the first regression, whilst rumination and numbing subscales remained significant in the final regression. Response to intrusions is thought to be a key posttraumatic variable in the maintenance of PTSD [21]. Theoretically and in practice, there is some overlap between the numbing subscale of the RIQ and the avoidance and numbing symptoms of the TEQ. However, although correlations between the subscales were significant they were low (*r*_*s*_ = .18). This may indicate that the RIQ numbing subscale identified responses that may not necessarily be indicative of PTSD symptoms. This is consistent with hypotheses put forward by Ayers and colleagues [43] who suggested that some predictors may be inflated by normal postnatal factors.

Deficits in intentional recall of the trauma memory were a significant predictor of PTSD symptom frequency. This is consistent with others [28] who found an association between memory disorganisation and PTSD symptoms. Many factors within childbirth may contribute to difficulties in memory that do not necessarily relate to finding the experience traumatic, such as pain and medications used to control pain [28]. However, results do suggest that intentional recall deficits are predictive of PTSD symptoms despite potential involvement of additional factors in the memory process. Surprisingly, low endorsement of negative appraisals of deficits in trauma memory was a predictor of increased PTSD symptom frequency. This finding is inconsistent with the positive correlation found with the variable in the sample and is not what would be expected. This result should be interpreted with caution, as the subscale only consisted of 3 items and was heavily skewed towards zero. It does suggest, however, that the impact of negative appraisals of recall deficits on PTSD symptoms is dependent on other variables included in the model, most likely intentional recall with which it is the most highly correlated.

Our study has some limitations, most notably the questionable representativeness of the sample recruited through the internet. The sample was entirely self-selecting and it is likely that those with an interest in research, reflecting on the experience of childbirth, or their mental health, were more likely to complete the survey. As such, any symptoms may have been inflated. It is not possible to know the nationality of the mother, although ethnicity was collected. This may have some implications in terms of the different health systems and cultural expectations about childbirth and mental health in different nationalities. In addition, we did not ask whether participants are first-time mothers or already have children, which might be a potential confounding variable. Secondly, the cross-sectional, retrospective design means that self-report of antenatal and birth-related variables may have been biased by the subsequent birth and postnatal experience or by current PTSD symptoms. This did not allow us to consider the role of the amount of time that had passed since childbirth, which is likely to influence some of the cognitive variables measured. Additionally, it is possible that the importance of post-trauma variables such as response to intrusions, cognitions and social support in the regression may be due to current symptoms being more sensitively measured than peritraumatic variables, largely due to the time that had elapsed since birth. Indeed, it has been reported that women’s memory of pain and birth experience can vary substantially between 2 months and a year [50]. It is possible that the self-report measures used for both risk factors and cognitive factors have a greater chance of association with PTSD symptoms due to shared method error variance. Additionally, it is possible that the scales measuring cognitive and behavioural factors are more likely to relate to measures of PTSD than objective antenatal risk factors, as they inherently describe similar, internal phenomena. Finally, the sole reliance on self-report questionnaires is another limitation, which might have led to the overreporting of symptoms.

The strengths of our study are a well-powered sample and the inclusion of different risk factors within one regression model. We comprehensively tested the validity of the most established theoretical model of PTSD in a postnatal population, including negative cognitive appraisals, dysfunctional cognitive and behavioural strategies, and trauma memory, all of which could potentially be targeted in the prevention or treatment of PTSD.

Our study has important clinical implications. Although demographic, obstetric and clinical risk factors are important, the predictive strength of cognitive behavioural variables when controlling for known risk factors suggests that the cognitive behavioural model provides significant additional value in furthering our understanding of PTSD symptoms following childbirth. The strength of the predictive value of negative cognitions about the self is of particular clinical importance; feelings of shame, self-blame, guilt and responsibility are therefore likely to be most valuable to identify and for which to provide a specific clinical intervention. Childbirth is a common event and is rarely experienced as traumatic; therefore it is important to be able to identify those women most at risk. Cognitive behavioural factors may also be more amenable to change postnatally than many of the risk factors previously identified in the literature [21].

The cognitive behavioural predictors that were identified as most significant are likely to be used already to inform clinical interventions for women experiencing PTSD following childbirth [24], although to date, only one intervention study offering CBT to mothers following premature birth has been published [51]. Our results confirm that the elements in the cognitive model are relevant to PTSD following childbirth and that CBT is likely to be clinically useful in this population. It may be that trauma-focused CBT interventions that support women to reduce negative cognitions of the self in relation to the childbirth, provide alternative responses to intrusions. In addition, work on deficits in intentional recall could be helpful. Furthermore, interventions based on CBT principles could also be built into postnatal groups for those most at risk, whilst also providing additional social support.

In terms of intra-partum clinical considerations, women’s perceived safety during childbirth appears important for the prediction of PTSD. It may be possible for medical staff to increase perceived safety in such situations, even if medical procedures are unavoidable. Additional qualitative research on the meaning of safety for women during childbirth may be beneficial in developing this finding further. Recent findings suggest that increasing psychological mindedness of the maternity service can in itself make a difference in terms of reducing trauma [52, 53]. Midwifery staff was provided with specific perinatal mental health training, and a communication system was introduced into the maternity services between psychology and midwifery. While preliminary, results suggested a significant decrease in the onset of PTSD symptoms following birth within this system.

Low positive social interaction and partner support during birth were significant predictors of PTSD symptoms. It is likely that increasing postnatal positive social interaction for those women at risk (e.g. through signposting to relevant groups or creating a buddy system) or a focus on how partners can best support women during birth (e.g. during birth preparation classes) could help to prevent the development of PTSD. Positive social interaction was also strongly correlated with other types of postnatal social support. Therefore, increasing these types of support is also likely to be beneficial for those at risk of developing PTSD. These findings should first be investigated prospectively to rule out the alternative interpretation that PTSD symptoms impacts on the perception of low support, rather than low support impacting on the development and maintenance of PTSD symptoms. However, if it is the appraisals of support that are associated with PTSD, then cognitive behavioural therapy may be a beneficial intervention.

Future research might usefully focus on prospective designs to identify how cognitive predictors relate to the development and maintenance of PTSD. Such studies would also allow for the inclusion of prospectively measured prior beliefs and coping and for investigation of the cognitive factors that predict longer term outcomes for those with traumatic stress reactions.

## Conclusions

This study showed that all variables derived from Ehlers and Clark’s cognitive model significantly explain variance in PTSD symptoms following childbirth, even when clinical, demographic and obstetric were controlled for. Our findings suggest that the CBT model is applicable and useful as a way of understanding and informing the treatment of PTSD following childbirth.
